# Extrinsic Factors Influencing Fetal Deformations and Intrauterine Growth Restriction

**DOI:** 10.1155/2012/750485

**Published:** 2012-07-19

**Authors:** Wendy Moh, John M. Graham, Isha Wadhawan, Pedro A. Sanchez-Lara

**Affiliations:** ^1^Center for Craniofacial Molecular Biology, Ostrow School of Dentistry and Children's Hospital Los Angeles, Keck School of Medicine of the University of Southern California, 4650 Sunset Boulevard, MS 90, Los Angeles, CA 90027, USA; ^2^Cedars-Sinai Medical Center, Medical Genetics Institute and David Geffen School of Medicine at UCLA, 8700 Beverly Boulevard, PACT Suite 400, Los Angeles, CA 90048, USA

## Abstract

The causes of intrauterine growth restriction (IUGR) are multifactorial with both intrinsic and extrinsic influences. While many studies focus on the intrinsic pathological causes, the possible long-term consequences resulting from extrinsic intrauterine physiological constraints merit additional consideration and further investigation. Infants with IUGR can exhibit early symmetric or late asymmetric growth abnormality patterns depending on the fetal stage of development, of which the latter is most common occurring in 70–80% of growth-restricted infants. Deformation is the consequence of extrinsic biomechanical factors interfering with normal growth, functioning, or positioning of the fetus in utero, typically arising during late gestation. Biomechanical forces play a critical role in the normal morphogenesis of most tissues. The magnitude and direction of force impact the form of the developing fetus, with a specific tissue response depending on its pliability and stage of development. Major uterine constraining factors include primigravida, small maternal size, uterine malformation, uterine fibromata, early pelvic engagement of the fetal head, aberrant fetal position, oligohydramnios, and multifetal gestation. Corrective mechanical forces similar to those that gave rise to the deformation to reshape the deformed structures are often used and should take advantage of the rapid postnatal growth to correct form.

## 1. Introduction

Intrauterine growth restriction (IUGR) is traditionally defined as a fetus who is at or below the 10th percentile in weight for its gestational age or an absolute birth weight of less than 2500 g [1]. Moreover, there is a pathological process present that prevents expression of normal growth potential, causing a decrease in fetal growth rate. Small for gestational age (SGA) describes infants with birth weights measuring less than the 10th percentile of standard growth curves [2]. Various criteria based on percentiles or standard deviations, including weight, length, and head circumference, have been developed to define this group, although many standards used are population-specific, and normal growth charts may have to be redefined [1]. It is important to distinguish between some small-for-gestational-age infants who are constitutionally small and, therefore, fall within the lower end of a normal distribution, but experience normal intrauterine growth and are not considered IUGR infants [3].

Fetal growth is sensitive to fetal environment, which is primarily determined by maternal physiology and placental function. Maternal birth size and infant birth size are correlated, showing a maternal intergenerational effect on birth size. Moreover, there is very low correlation for birth weight of half siblings with the same father, but different mothers [4]. In a study of rhesus monkeys through several generations, Price and Coe found that the mother's gestational experience can influence the intrauterine environment she provides for her own offspring. Maternal half-brothers and half-sisters had similar birth weights, while paternal half-brothers and half-sisters had less of a deviation from average-for-date birth weights. Intrauterine constraint probably reflects altered maternal metabolic processes or uterine/placental transport mechanisms that limit the provision of nutrients to the fetus. There exists a degree of gestational imprinting between mothers and daughters during fetal development [5]. Barker has hypothesized that nutritional and other environmental cues during development can permanently alter the structure, homeostatic systems, and functions of the developing fetus and affect adult cardiovascular and renal function [6]. This effect, which is termed “fetal programming,” has been investigated by epidemiological and animal studies that suggest that programmed effects operate within the normal range of growth and development, and impact the risk for hypertension in adult life, but this effect will not be further discussed in this paper, which will discuss other fetal environmental effects.

It has also been shown that short-term maternal undernutrition leads to an immediate slowing of fetal growth. Embryonic growth and fetal size are ultimately determined by the interplay of supply of nutrients to the fetus by the uteroplacental unit and the fetal endocrine/paracrine status. Maternal endocrine factors also influence the supply of nutrients. Various systems have evolved to give priority to mother over fetus, viewing the fetus as replaceable. Maternal constraint describes the set of nongenetic and nonpathological influences through which the mother limits fetal growth, specifically, the absolute limitation on the capacity of the mother and placenta to supply nutrients to the fetus [4].

Maternal constraint has received relatively minor biomedical consideration being physiological rather than pathological, although its effects are well recognized in the epidemiological studies of birth weight. However, recent recognition that changes within the normal range of fetal development can have long-term consequences for the risk of disease indicate that these physiologically constraining influences deserve more attention [4]. 

## 2. Normal Model for Birth Size

In typically developing singleton fetuses, there is little variation in fetal growth up to 16 weeks of gestation. Considerable variance occurs during the mid- and late-gestation periods [7]. Exclusive of chromosomal and genetic abnormalities, the predominant cause of fetal growth retardation correlates to diminished supply of nutrients. Maternal habitus and physiology largely influences birth size, showing an association between height, uterine size, and blood flow [1, 8].

Normal intrauterine growth occurs in three stages. The first stage takes place during 4–20 weeks of gestation, rapid cell division and multiplication (hyperplasia) occurs as the embryo grows into a fetus. The second stage, 20–28 weeks of gestation, cell division declines and the cells increase in size. The third stage, 28–40 weeks, there is rapid increase in cell size, rapid accumulation of fat, muscle, and connective tissue. Most fetal weight gain occurs during the last 20 weeks of gestation. If during this delicate time of development and weight gain is disturbed or interrupted, the baby can suffer from IUGR [9].

## 3. Symmetric versus Asymmetric IUGR

There are two general patterns of growth abnormalities: symmetric and asymmetric. Symmetric growth inhibition arises during the first stage, when fetal growth occurs primarily through cellular division and produces an undersized fetus with fewer cells of normal size. Characterized by a proportional lack of growth, including smaller dimensions in skeletal size, head size, and abdominal circumference, the weight, head, and length are all below the 10th percentile [3]. This early reduction in fetal cellular proliferation occurs in 20–30% of IUGR infants [2]. The cause is thought to be an early intrinsic impairment, such as chromosomal abnormalities and congenital malformations, drugs and other chemical agents, infection, or maternal metabolic disorders.

Conversely, asymmetric growth inhibition occurs during stages two and three of gestation and is usually the consequence of an inadequate availability of substrates for fetal metabolism. This pattern results in a decrease of cell size and fetal weight with less effect on total cell number, fetal length, and head circumference. Asymmetric growth restriction is the most common form and occurs in 70–80% of IUGR infants [2]. The musculoskeletal dimensions and head circumference are spared, and the abdominal circumference is decreased due to subnormal liver size and a paucity of subcutaneous fat. The most common disorders that limit fetal metabolic substrate availability are maternal vascular disease and decreased uteroplacental perfusion, which generally present later in pregnancy when fetal growth occurs primarily by an increase in cell size rather than cell number [3].

Distinguishing between the disparate causes of symmetric and asymmetric growth can provide useful information for diagnostic and counseling purposes. A diagnosis of asymmetric IUGR early in pregnancy may suggest a poor prognosis when considering the various etiologies, while asymmetric IUGR during the third trimester may carry a more optimistic prognosis with careful medical management. Symmetric IUGR with a normal interval rate of growth may simply represent a constitutionally small, but otherwise normal fetus, or it could be due to one of many genetic primordial short stature syndromes if both parents manifest normal growth [3]. 

## 4. Types of Congenital Anomalies

There are four main types of congenital anomalies, which include malformations, disruptions, deformations, and dysplasias (Table 1). The main focus of this paper concern deformations, which are abnormalities of form or position of a part of the body caused by nondisruptive mechanical forces [10]. Deformation is the consequence of extrinsic biomechanical factors (Table 2), where the pliable growing fetal tissues are molded in response to the aberrant constraint, and should be distinguished from malformation where there is an intrinsic problem in one or more of the developing tissues of the fetus [11]. Disruptions represent the breakdown of previously normal tissues. Dysplasias result in abnormal structure because the tissues from which individual structures are formed are abnormal [12].

Deformation can result from mechanical interference with the normal growth, functioning, or positioning of the fetus in utero. About 2% of babies are born with an extrinsic deformation, 90% of which resolve spontaneously, making them relatively common problems [13]. Extrinsic forces may result in a single, localized deformation, such as a positional foot deformity, or they may cause a deformation sequence, referring to the manifold molding effects of a given deforming situation. Uterine constraint of the rapidly growing, malleable fetus in late gestation is the major cause of deformations, when the size of the fetus is large in comparison to the size of the uterus [12]. 

Prior to 36-37 weeks of gestation, the amount of amniotic fluid is usually adequate to cushion the fetus and allows for normal growth and mobility. One of the major functions of amniotic fluid is to distend the uterus, thereby enabling the fetus to move freely and grow with equal pressure in all regions without excessive or localized constraint [14]. During late gestation when the fetus becomes more crowded within the uterus, it will usually settle into a position where the largest moving fetal parts, the bulkier legs, have the most room, in the upper portion of the uterus, assuming a vertex presentation. After 35–38 weeks of gestation, the fetus becomes increasingly constrained as it tends to grow out of proportion to the size of the uterine cavity. The relative proportion of amniotic fluid decreases during this period of rapid late fetal growth, contributing to uterine constraint [12].

Constraining factors include first pregnancy, small maternal size, small uterus, uterine malformation, large uterine fibroids, small maternal pelvis, early engagement of the fetal head into the mother's pelvis, unusual fetal position, oligohydramnios, large fetus, and multifetal gestation. As these abnormalities are caused by mechanical factors, they can often be treated by mechanical means [10]. Most of these molded deformations have an excellent prognosis once the fetus is released from the constraining environment; however, prompt treatment shortly after birth is necessary to achieve the best results. Treatments involve the use of gentle mechanical forces to reshape the deformed structure into a more normal form. When fetal growth has been constrained during late gestation, catch-up growth is usually initiated promptly after birth with most term fetuses reaching their genetically determined growth percentiles in 6–8 months [12].

## 5. Biomechanics

There are simple basic principles relative to deformation. The magnitude and direction of the forces impacting the form of the developing fetus and the response of a given tissue depend on its pliability and stage of development. Some factors that affect the magnitude and direction of mechanical forces include external resistance to growth and/or movement, growth rate and shape of basic tissue, forces of fluid flow or pressure, plasticity of the fetus, forces of muscle pull, and forces of gravity. There is an integral interaction between growth and the forces of tension and compression evident in the relationship between muscle usage and muscle mass size. The greater the usage, the larger the muscle mass tends to become. Moreover, the stress of muscle tendon pull on a bone affects its growth and formation. With increased muscular pull, there is increased size of the bony promontory at the site of muscle attachment on the bone. Biomechanical forces play an important and specific role in the normal morphogenesis of most tissues, with certain tissues having their own limited repertoire of responses to forces [12].

## 6. Primigravida

The first fetus must initially distend the mother's uterus and abdominal wall during the first pregnancy when the uterus is more resistant to stretch. Consequently, the first-born infant usually experiences more constraint and is usually smaller than the second or third by about 200–300 grams, although they are of comparable size by one year of age [15]. Evolutionarily, a mother must limit her investment in the first child in order to survive, not only to nurture that child, but also to have subsequent children [1]. The first-born infant is also more likely to become constrained in an unusual position and thereby have consequent deformations relating to malpresentation [12]. In pregnancies with nonimpaired placental perfusion, normal presentation and normal amounts of amniotic fluid, extrinsic forces and associated deformations are less likely. 

## 7. Small Maternal Size

Deformations are more common in offspring born to smaller women than in larger women. The smaller the mother's size in relation to the size of the fetus, the greater the likelihood of deforming uterine constraint in late fetal life. Maternal size has a much greater impact on birth size than paternal size, although the length of the infant equally relates to maternal and paternal stature by one year of age. This transient effect on birth size appears to relate to small maternal size in restraining late fetal growth [12].

In the classic Walton and Hammond cross-breed study of Shire horses and Shetland ponies, maternal influences on growth and conformation were observed. Maternal phenotype was found to determine the size of the fetus. In their cross-breed experiments, they found the fetus at birth to be approximately proportional to and regulated by the size of the mother. Although, the genetic differences appear shortly after the maternal regulation is withdrawn, and growth is proportional to the normal size of the genotype. The size of the young is not limited to the size of the uterus, but correlated to the size of the placenta. Being an organ of the fetus, the placenta varies with fetal weight and is affected by the same changes as the fetus itself. The mother's size determines the level of nutrients available, which in turn controls the growth of the fetus. This is also demonstrated by the fact that individual young in multiple births are smaller in single births due to competition for a common food supply [16].

There is also a very strong correlation between placental weight and birth weight, which remains relatively constant even in pathological circumstances, such as maternal anemia. Although this proportional relationship holds true, it is unclear whether there is a primary effect of placental weight on fetal growth or whether it is simply to the common genotype and environment of the two systems. In an experimental study on mice, differences among immunized, tolerant, and control mothers were more pronounced for placental weight than birth weight, suggesting that fetal growth was secondarily affected. Furthermore, removal of part of the placenta in this study was shown to have reduced fetal growth [17].

## 8. Uterine Malformation

An estimated 1-2% of women have a clinically significant malformation of the uterus posing a 30% general risk of fetal deformation [18]. A malformed uterus due to failure of the mullerian ducts to completely fuse during embryogenesis can result in either symmetric or asymmetric structural anomalies such as didelphic uterus with duplicated cervix, bicornuate uterus, septate uterus, and arcuate uterus. Associated deformations include craniofacial deformations, overlapping sutures, joint contractures, limb deformations or disruptions, edema or grooves, and thoracic constriction resulting in pulmonary hypoplasia. Severe uterine constriction can also lead to vascular disruption within the fetal limb [12].

All gradations of fetal deformation may occur in mothers with a uterine cavity deformation. Compression from the septum of a bicornuate uterus can attribute to grooves or depressions in developing fetus [19]. Prolonged constraint within a bicornuate uterus can result in multiple joint contractures from fetal immobility, but these usually resolve with physical therapy [20]. Documented reports of small uterine cavity births have presented with grooves or depressions in the compressed area of the fetus, which resolve or improve postnatally. Constraint of thoracic growth could be severe enough to impair lung growth and maturation causing pulmonary hypoplasia. Mullerian duct anomalies may be familial, often in association with renal and urinary tract anomalies, and should be further investigated [21–23].

In a case control study of 38 infants (32 livebirths and 6 stillborn) born to mothers with a bicornuate uterus, there was fourfold greater risk of congenital defects compared to mothers with a normal uterus [24]. There were five defects that were significantly more common including nasal hypoplasia, omphalocele, limb deficiencies, teratoma, and acardiaanencephaly. In this study, 13 of the 38 women with bicornuate uteruses had deformations. There was a significantly higher incidence of vaginal bleeding in women with uterine malformations than in women with a normal uterus, 54.1% versus 14.1%, respectively, with vaginal bleeding being associated with limb defects [24, 25].

Many genetic factors that result in bicornuate uterus also affect renal morphogenesis, leading to renal dysplasia and resultant oligohydramnios and further increasing the chances of fetal deformation [12]. Surgical correction to improve the uterine size or shape may greatly increase the chances of rearing a normal fetus to term birth, providing a better opportunity for the fetus to grow without as much constraint [26]. 

## 9. Uterine Fibromata

A large uterine fibroid may limit intrauterine space and can result in fetal deformation similar to that of a bicornuate uterus [25]. Most uterine fibromata develop relatively late in reproductive life and are an infrequent cause of fetal deformation, however, sometimes increased levels of maternal estrogen during late gestation can rapidly enlarge a small fibroma. Depending on maternal age, uterine myomata occurs in 2–15% of pregnancies. While most myomas remain small and asymptomatic, 10–40% of affected pregnancies will have myoma-related complications [27–29]. 

## 10. Small Maternal Pelvis

Appreciable molding of the craniofacies may result from vaginal delivery through a pelvic outlet that is small relative to the size of the fetus. Although this molding is usually transient, if there has been prolonged engagement of the fetal head, the degree of molding could be severe and there may be a slower resolution toward normal form after birth. This may be facilitated by early treatment with appropriate positioning [12].

## 11. Early Pelvic Engagement of the Fetal Head

During labor, as the fetal head enters and passes through the pelvic inlet, it usually engages in an occiput transverse (OT) position. The left-oriented occiput position is more common than the right-oriented occiput position, 58.5% and 40.5%, respectively, [30, 31]. As the fetal head progressively descends, it traverses the pelvic inlet with the saggittal suture in the transverse diameter and the biparietal diameter (BPD) parallel to the anteroposterior diameter of the pelvic inlet. Internal rotation also occurs, usually passing the ischial spines in an occiput anterior or posterior position. There is further flexing of the fetal head as it encounters resistance from the cervix, pelvis walls, and pelvic floor. Engagement of the fetal head occurs when the BPD, the largest transverse diameter of the fetal head, has traversed the pelvic inlet. Fetal head descent usually takes place shortly before birth. In 58.5% of cases, the head in vertex presentation rotates into the left occiput transverse position in order to pass through the mother's pelvis [12].

Early engagement of the fetal head is a rare event and often accompanied by maternal symptoms of marked pelvic pressure and pubic discomfort, with pain sometimes radiating down the back of the legs. Descent of the fetal head rarely occurs more than one month before delivery, although more common in the primigravida. The fetal head is a relatively large and rapidly growing structure, making it especially susceptible to deformation. The most common problem resulting from early engagement is congenital muscular torticollis, which can lead to plagiocephaly without appropriate therapy. Other potential consequences include vertex craniotabes secondary to prolonged compression of the top of the calvarium [32], lateral constraint of the fetal head leading to lack of growth across a given suture and craniosynostosis [33], and transient vertex molding that usually resolves within a few days [12].

## 12. Fetal Position

Prior to 36-37 weeks of gestation, the fetus has adequate room for movement and is commonly found in varying positions, particularly breech presentation. As the fetus grows larger and becomes more crowded, it tends to shift into vertex presentation, where the bulkier legs have more room in the upper portion of the uterus. The most prevalent cause for an aberrant position is fetal constraint that has a limited capacity to move into vertex presentation, which could significantly affect craniofacial structures [12].

## 13. Breech Presentation Deformation

One-third of all extrinsic deformations occur in babies who have been in breech presentation, as found in Dunn's early studies on congenital deformations [13, 34]. Breech presentation occurs when the buttocks or legs of the fetus are the first parts to appear at the uterine cervix during delivery. Numerous fetal and maternal factors lead to this presentation including prematurity (25% breech), twinning (34% breech), oligohydramnios (64% breech), uterine malformations, placenta previa, maternal hypertension, and fetal malformations [12].

Breech presentation has a familial tendency, with 22% of multiparas having previously experienced a breech presentation delivery. This may be due to inherited uterine structural characteristics, or it may be consequent of a genetic neuromuscular or fetal malformation syndrome [12].

In 70% of breech presentation cases, the fetus has its legs extended in front of the abdomen, limiting fetal movement and reducing the chances of releasing itself from this position [35]. Prolonged breech position during late gestation results in the “breech head” of dolichocephaly, anteroposterior elongation of the head, with a prominent occipital shelf [36]. The mandible may be distorted, and the shoulders thrust under the lower auricle [12].

Most studies show the risk for neonatal mortality and morbidity is increased when breech infants are delivered vaginally, versus cesarean section. The mechanism of injury in vaginal breech delivery is usually longitudinal distraction and occurs most frequently in the lower cervical or upper thoracic region. Although the vertebral column is left intact, this results in symptoms of diaphragmatic breathing and hypotonia with hyperreflexia that is worse in the lower extremities, with possible associated brachial plexus and phrenic nerve injury. [37, 38] Other traumatic injuries that can result from vaginal delivery of infants in breech presentation include fractures and dislocations, brachial plexus injuries, facial nerve injuries, cerebral hemorrhages, bruising with hyperbilirubinemia, cervical cord injuries, cord prolapse, birth asphyxia, and testicular trauma [12].

Three factors must be considered in the management of breech presentation. First, prevent deformities and complications from vaginal birth by using the external cephalic version method, externally manipulating the fetus into vertex position before the time of delivery. This is usually accomplished between 35 and 37 weeks from conception with a 79% success rate [39]. Performed before 35 weeks, the fetus may revert to breech presentation. After 37 weeks, external version is more difficult to accomplish. Even so, performed after 38 weeks, the procedure is 40% successful in nulliparous women, using tocolytics to relax the uterus, and 60% successful in multiparous women [40]. Second, avoid complications related to vaginal delivery by using cesarean section delivery, particularly if the head is hyperextended. Factors that favor cesarean section delivery of a fetus in breech presentation, thereby forestalling the potential vaginal delivery complications, include dolichocephalic breech head, contracted pelvis, placenta previa, maternal hypertension, uterine dysfunction, elder primigravida, and previous pregnancy losses [41–43]. Third, manage any deformations and complications after delivery of the breech fetus. Generally, head shape and mandibular form naturally resolve to normal form postnatally, hip dislocation and hydrocele of the testis may indicate more rigorous management or therapy. Sternocleidomastoid muscles tears may occur, with 20% of torticollis cases occurring in breech infants [44]. Early treatment of torticollis can prevent secondary plagiocephaly [12].

## 14. Transverse Lie Deformation

Occurring in 2.5 per 1000 deliveries, transverse lie occurs when the long axis of the fetus is perpendicular to that of the mother. It is often associated with multiparity (90%), prematurity (13%), placenta previa (11%), polyhydramnios (8%), uterine anomalies (8%), and uterine myomas (3%) [45]. Multiparity is the most common factor; laxity of the abdominal musculature accounts for the liability toward transverse lie in these women. Polyhydramnios occurrence may be accounted for due to the fetus being unable to swallow amniotic fluid since the mouth is pushed up against the side of the uterus. The remaining associations occur more frequently with primiparas [12].

Features that may present in the case of full frontal constraint include a flattening of the face, limited mandibular growth, and a retroflexed head with a prominent occipital shelf. There may also be other associated deformations, such as torticollis and/or scoliosis. Transverse lie at term can be managed by external cephalic version or elective cesarean section. With expectant management, there is an 83% spontaneous conversion rate to longitudinal lie before labor [46, 47]. Although, there is an increased risk of cord prelapse and birth trauma/asphyxia with persistent transverse lie. External version ensued by induced labor is recommended for multiparous women since laxity of the abdomen is the major cause, but primiparous women should not be treated in the same way as their condition is likely to be accounted for by some kind of underlying pathology [47]. A study comparing management of unstable lie in late pregnancy found that when managed with external version, 94% of multiparous women had successful vaginal deliveries, whereas 44% of primiparous women required emergency cesarean sections [48]. When unresolved, transverse lie can result in uterine rupture and other complications [49, 50]. Therefore, it should be managed through cesarean section using a lower uterine segment incision [12].

## 15. Face and Brow Presentation Deformation

Face presentation, 1-2 per 1250 deliveries, is slightly more common than persistent brow presentation, 1 per 1444 deliveries [51–53]. High parity and cephalopelvic disproportion have been proposed as etiologic factors in face and brow presentation. In face presentation, the fetal head is hyperextended with the occiput touching the back. The presenting part is the fetal face between the orbital ridges and the chin. Mostly diagnosed by vaginal examination during delivery, face presentations are 59% mentum anterior, 15% mentum transverse, and 27% mentum posterior [53]. Due to compression of the chin and retroflexion of the neck, redundant skin folds in the anterior upper neck with persistent retrognathia and a prominent occipital shelf may be present. In more severe cases, there are associated feeding difficulties with trouble swallowing or jaw subluxation with an audible clicking as the jaw moves in and out of its socket [12].

In brow presentation, the fetal head is between flexion and hyperextension. The presenting part is the fetal brow between the anterior fontanelle and orbital ridges [53]. Many persistent brow presentations are associated with cephalopelvic disproportion and result in prolonged dysfunctional labors. The fetal mouth may be forced open as the jaw is pushed against the fetal chest, lengthening the presenting diameter. This could lead to congenital jaw subluxation with audible clicking as the jaw moves in and out of the temporomandibular joint socket. The brow is abnormally prominent, while the midface is less prominent than normal. There may be caput succedaneum in the frontoposterior position, making conversion more difficult and prolonging labor in 40–50% of cases [53–55].

Face and brow presentation increase the risk of difficult labor, with vaginal delivery being possible only for mentum anterior face positions due to maximum extension of the fetal neck when in the posterior position. Although, many fetuses with mentum posterior position will spontaneously convert into mentum anterior position upon reaching the vaginal floor, thus allowing for vaginal delivery [53]. Cesarean section is considered particularly if the fetus is large, the mother's pelvis is small, or there is a persistent mentum posterior positioning with arrested descent. Prolonged compression of the neck against the pubic ramus during delivery could cause damage to the trachea or larynx [56].

Postnatal catch-up growth of the infant usually occurs after delivery with face and brow presentation deliveries, which usually resolve the abnormalities. The restrained jaw begins to grow toward normal, the head gradually resumes a more normal posture, and the redundant skin folds on the anterior neck resolve with time. Congenital jaw subluxation requires only gentle massage. There is no significant recurrence risk [12].

## 16. Oligohydramnios

Oligohydramnios is a serious deficiency of amniotic fluid, which results in significant fetal constraint. This condition may be secondary to amniotic rupture and may be accompanied by constrictive, disruptive strands of amnion. Early amnion rupture can lead to compressive consequences of early constraint including scoliosis and clubfeet, in addition to vascular disruptions leading to facial clefts and limb reduction with body wall defects. The most lethal consequence is spontaneous abortion. Late amnion rupture may be accompanied by amnion bands, usually limited to constrictive bands around various parts of one or more limbs [12]. Common causes of oligohydramnios include amniotic fluid leak and decreased amniotic fluid production due to placental hypoperfusion or fetal anuria. Features of this sequence include pulmonary hypoplasia, positional deformities of the hands and feet, and Potter's faces [10, 57]. The major source of amniotic fluid is fetal urination, of which 1000–1200 mL enters the amniotic space each day from the fetal kidneys, which begin to develop around 10–12 weeks of gestation. Additional fluid also enters from the fetal lungs. Complete turnover of amniotic fluid volume, about 800 mL at term, occurs in less than 24 hours. Around 500 mL is swallowed each day, with additional fluid moving into the maternal and fetal circulation through vessels in the amniotic membranes and placenta [58].

Adverse perinatal outcomes associated with this condition are fetal (25%) and/or neonatal (31%) acidosis, fetal heart rate abnormalities, low Apgar scores, meconium staining (29%), and fetal distress requiring emergency cesarean section (64%) [59, 60]. Lack of adequate urine flow into the amniotic space may be caused by renal agenesis, renal dysfunction, or fetal urinary tract abnormalities. Renal agenesis is the most common cause, but polycystic or multicystic dysplastic kidneys or obstructive uropathy may also be associated with oligohydramnios [61]. The amount of amniotic fluid tends to decrease during the last trimester as the fetus fills out the uterine cavity. Fetal immobilization during late gestation from oligohydramnios is associated with positional limb abnormalities, although not reduced bone mass, suggesting muscular stress is a major factor in fetal periosteal bone growth [62]. Poor placental function leads to decreased fetal hydration, growth retardation, and decreased fetal urinary flow [12].

Amniotic fluid volume rises progressively from 10–20 mL at 10 weeks to 800 mL at 24 weeks, then remaining relatively constant until term [63, 64]. After 40 weeks, amniotic fluid volume declines by about 8% per week. Oligohydramnios is considered approximately 300 mL, the 5th percentile for gestational age [58]. Persistent oligohydramnios may be an indication for prompt delivery. Perinatal mortality rises 13-fold when sonographic fluid volume is marginal and 47-fold with severe oligohydramnios [58]. Features of this condition are similar to those of breech presentation, as about 50% of cases are in breech presentation at birth due to their inability to undergo normal version in late gestation. Oligohydramnios tetrad describes the main features in nonrenal cases, which are facial compression, aberrant hand and foot positioning, fetal growth deficiency, and pulmonary hypoplasia [57]. Although recent studies have found that premature rupture of membranes does not increase risk for growth deficiency in fetuses with oligohydramnios [59, 65–67].

Craniofacial features appear as though a silk stocking has been pulled over the head, causing a flattened nose and the appearance of low-set, flattened, and enlarged external auricles [12]. Limb defects include edema of the hands and feet with positional deformation and stiffness of the joints with flexion contractures of the elbows, knees, and feet [68]. Fortunately, limb defects of surviving infants respond readily to physical therapy. Thoracic growth is restrained due to inhibition of breathing movements essential for lung growth and/or abnormal fluid dynamics within the lungs themselves resulting in decreased intraluminal pressures [69]. External constraint leads to overgrowth of skin giving rise to accentuated inner canthal and infraorbital skin folds. This redundant skin characterizes the “Potter faces” and yields a false impression of a webbed neck [70].

The gestational age of onset and the duration of severe oligohydramnios are independent risk factors. When membrane rupture occurs at an early gestational age, there is a predisposition to joint contracture and the risk of deformation relates to the duration of the oligohydramnios [68]. Surviving infants usually experience catch-up growth within a few months of birth and show restitution toward normal growth form with physical therapy. Rupture before 24 weeks poses a great risk for pulmonary hypoplasia making prompt delivery untenable. Rupture after 24 weeks does not increase the risk of pulmonary hypoplasia, but can affect the development of skeletal deformation. Therefore, once the risk of prematurity complications becomes low, delivery within two weeks of rupture appears feasible [71]. Other measures include efforts to increase amniotic fluid volume through maternal bed rest or instillation of antibiotics and saline into the amniotic cavity [72, 73].

Recurrence risk of oligohydramnios in subsequent pregnancies varies with the basic problem that led to the condition. With chronic leakage of fluid, the risk is generally very low. Defects such as renal agenesis or obstructive uropathy have a higher risk of recurrence and renal anomalies in first-degree relatives [74]. The risk may be as high as 25% with infantile polycystic kidney disease due to autosomal recessive inheritance [61]. When oligohydramnios is due to a fetal malformation problem, prenatal diagnosis should be offered for subsequent pregnancies [12].

## 17. Large Fetus with Rapid Growth

Normally doubling in weight between 28–34 weeks of gestation, the fetus exhibits a rapid rate of growth. The faster the growth rate and larger the fetus, the more likely the chance of all types of external constraint-related deformations. Many extrinsic deformations are more common in the male than in the female, as males are normally larger and grow more rapidly in late fetal life [75]. One exception more common in females is developmental dysplasia of the hip and other similar joint-dislocation deformations that appear to be related to greater connective tissue laxity in females. The effects of relaxing hormones and their receptors in females may allow for cervical dilation, increasing their susceptibility to congenital hip dislocation and protecting them from torticollis. Conversely, testosterone may accentuate muscular development in males, protecting them from hip dislocation and increasing their susceptibility to torticollis [12].

## 18. Multifetal Gestation

Multifetal pregnancies compounds the incidence of asymmetric IUGR by 65–85% compared to singleton pregnancies, resulting in dramatically increased morbidity to the fetus. Two important ways the placenta may adapt to increased fetal requirements seen throughout pregnancy include increased placental weight and changes in morphology. In a study comparing placental changes with uterine space restriction, total placental weight was increased by 68% and 120% in twin and triplet pregnancies, respectively, compared to singleton pregnancies. Even as placental adaptations occur, fetal growth arrest leading to asymmetric IUGR is still observed. These changes in placental efficiency are critical in preserving a viable albeit compromised fetus [76].

The average uterus is capable of handling a maximum of 4 kg of fetal mass. Multiple fetuses fill out the uterine cavity more quickly than a singleton fetus. Twins usually fill the uterine cavity around 34 weeks, after which growth slows as it becomes crowded [77]. Transient growth deficiency and postural deformations are more common in twins, especially malpositioning of the feet and molding of the cranium [77–80]. Particularly frequent in multiple births, torticollis-plagiocephaly and secondary craniofacial deformations usually affect the bottom-most twin. Since one twin may have been constrained to a different extent than the other, monozygotic twins may not appear identical at the time of birth [12].

Multiple gestations are associated with both growth restriction and preterm delivery. After 32 weeks of gestation, the growth curve of twins deviates from that of singletons, and 15–30% of twin gestations may be growth-restricted [3]. Late fetal crowding makes deformations more common in twins. Deformations are equally likely in monozygotic and dizygotic twinning. Monozygotic twins often share the same placenta and placental vasculature, making them more vulnerable to vascular disruption [12]. Although IUGR can occur in both, monochorionic twins are at greater risk for growth issues and subsequent long-term complications compared to dichorionic twins. Monochorionic twins share a placenta and interplacental vascular anastomoses, which can lead to discordant growth due to unequal placental sharing, placental cord abnormalities, and twin-twin transfusion syndrome (TTTS). Greater than 20% of monochorionic diamniotic twins were found to have growth discordance, with about 15% developing TTTS. Severe growth abnormalities in dichorionic twins are usually diagnosed in the third trimester and affect one twin, placing the parents in a difficult position. Management choices depend on the etiology of the growth issue, the severity of the problem, duration of growth discordance, and gestational age at diagnosis. Options include expectant management, termination of the entire pregnancy, and selective termination of the IUGR fetus [81]. 

## 19. Management and Prevention

Management of extrinsic deformation varies with the cause and type of anomaly, although early treatment is always critical to a successful outcome. Accurate diagnosis of growth restriction, beyond fetal size alone, is enhanced by integration of other indicators of fetal and placental health. Commonly used clinical tools include antenatal testing for fetal health and for placental function. Evaluation of fetal health includes fetal heart rate analysis, amniotic fluid volume assessment, biophysical profile, and Doppler fetal and maternal vessel evaluation. Although there are limited studies for predicting IUGR, the combination of maternal plasma biochemistry markers with second-trimester uterine artery Doppler measures appear promising for prediction. New developments in genetic epidemiology could identify DNA polymorphisms that provide additional markers to improve the predictive ability of screening and diagnostic tests in identifying fetuses at risk of abnormal fetal growth [82].

In an otherwise normal infant, when deformation is due to external constraint in late fetal life, there is usually an excellent prognosis for return to normal form. It may be worthwhile to observe the neonate for several days before determining whether any further therapy is required, as spontaneous changes could occur. Treatment often uses corrective mechanical forces similar to those that gave rise to the deformation to reshape the deformed structures into a more normal form [12]. Mechanical therapy should also take advantage of the rapid postnatal growth to correct form. This is especially beneficial for the torticollis-plagiocephaly deformation sequence, which must be corrected with physical therapy of the neck and repositioning of the infant's head in a timely manner. Use of orthotic management when the head is still rapidly growing has consistently been documented to correct deformational cranial asymmetry [80, 83, 84].

Precise management methods for constraint-related deformations may vary appreciably, from benign measures to rigorous molding. Deformation due to external constraint in late fetal life, in an otherwise normal infant, has an excellent prognosis for return to normal form. Simple daily manual manipulation of molding and stretching toward a normal form is common practice in India. Reshaping of foot deformations can be gradually improved through forces of frequent adhesive taping and molding. When such gentle measures do not correct the deformation, casting of the limbs or orthotic molding of the head may be used [80, 83, 84]. Surgical intervention may be indicated for seriously dislocated or malpositioned joints that cannot be corrected by more conservative measures. Earlier surgery is especially important for developmental dysplasia of the hip and severe equinovarus deformity of the calcaneus and talus. Proper bony alignment is required to foster subsequent normal joint development [12].

Several preventive measures already exist in medicine today. Surgical repair of a malformed uterus that had previously resulted in serious deformation problems and decreased fetal survival may greatly improve prognosis for a normal offspring. In the case of oligohydramnios, there may be some benefits gained from restoring amniotic fluid volume with saline or amnioinfusion with a similar fluid following preterm premature rupture of the membranes (PPROM). Although benefits in growth and prevention of fetal deformation have not been studied, benefits may include preventing infection, lung damage, and death of the baby as well as by preventing infection of the womb after childbirth in the mother. However, there is currently insufficient evidence to recommend routine use of amnioinfusion for PPROM [85]. Fetal surgery for diaphragmatic hernia urethral obstruction malformation sequence may improve fetal viability. External cephalic version for a fetus in abnormal presentation is now an accepted method in medicine. Prenatal detection and management may prevent serious birth trauma [12]. Research on modifiable methods to reduce or prevent extrinsic intrauterine physiological constraint merits additional consideration and further investigation.

## Figures and Tables

**Table 1:** Types of congenital anomalies.

Malformations	Disruptions	Deformations	Dysplasias
(i) Morphologic defects resulting from intrinsically abnormal developmental processes	(i) Breakdown of, or interference with, an originally normal developmental process	(i) Abnormalities of form or position of a part of the body caused by nondisruptiveme chanical forces	(i) Abnormal structure because the tissues, from which individual structures are formed, are abnormal
(ii) Occur early in embryogenesis	(ii) May occur at any time during gestation	(ii) Usually develop during the second half of pregnancy	(ii) Often due to single abnormal genes

**Table 2:** 

Possible extrinsic causes of deformation
(i) Primigravida
(ii) Small maternal size
(iii) Uterine malformation
(iv) Uterine fibromata
(v) Small maternal pelvis
(vi) Early engagement of fetal head
(vii) Unusual fetal position
(viii) Oligohydramnios
(ix) Large fetus with rapid growth
(x) Multifetal gestation
